# Anaphylaxis in Children: Current Understanding and Key Issues in Diagnosis and Treatment

**DOI:** 10.1007/s11882-012-0284-1

**Published:** 2012-07-20

**Authors:** Chitra Dinakar

**Affiliations:** Section of Allergy, Asthma and Immunology, Children’s Mercy Hospital and Clinics, University of Missouri-Kansas City, 2401 Gillham Road, Kansas City, MO 64108 USA

**Keywords:** Epinephrine, Food allergy, Food hypersensitivity, Pediatric, Children, Anaphylaxis, Diagnosis, Treatment

## Abstract

Anaphylaxis is a severe allergic reaction that is rapid in onset and may cause death. Since it is unpredictable and potentially fatal, prompt recognition and treatment are vital to maximize a positive outcome. The occurrence of anaphylaxis is increasing across all ages in the United States, with increased risk of worse outcome in teenagers/young adults and in those with comorbid conditions such as asthma. Gaps in the assessment of patient-specific risk factors, identification and prevention of triggers, recognition of signs/symptoms, and pharmacologic treatment of anaphylaxis have been identified at the physician and caregiver/patient level. A PubMed literature search (January 2000–December 2011) was conducted to identify publications on childhood anaphylaxis using the following terms: food allergy, food allergens, food hypersensitivity, epinephrine, epinephrine auto-injectors, anaphylactic triggers, and anaphylaxis. This review will critically appraise these key issues and highlight strategies that might result in improved management of anaphylaxis in children.

## Introduction

Anaphylaxis is a potentially fatal condition that can occur without warning [1]. Prompt diagnosis and treatment are crucial [2••, 3••]. Previously, the lack of a universal definition for anaphylaxis resulted in misdiagnosis, underreporting, and miscoding, impeding epidemiological research on this condition. To address this issue, a definition was standardized in 2005 by the Joint Task Force on Practice Parameters, representing the American Academy of Allergy, Asthma and Immunology (AAAAI), the American College of Allergy, Asthma and Immunology (ACAAI), and the Joint Council of Allergy, Asthma and Immunology (JCAAI) [4]. The Joint Task Force defined anaphylaxis as “a condition caused by an [immunoglobulin E] IgE-mediated reaction” that is “often life-threatening and almost always unanticipated.” Anaphylactoid reactions were defined as non-IgE-mediated reactions with the same clinical picture as anaphylaxis. When both IgE-mediated and non-IgE-mediated mechanisms were a possible cause, the term anaphylactic was used to describe the reaction.

Due to the wide variability in defining anaphylaxis, incidence and prevalence data should be interpreted with caution [5]. In a population-based study in Rochester, Minnesota from 1990–2000, the annual age- and sex-adjusted incidence of anaphylaxis was estimated to be 49.8 per 100,000 person-years [6]. In this study, age-specific rates were highest for ages 0–9 years (75.1 per 100,000 person-years) and 10–19 years (65.2 per 100,000 person-years). In contrast, a previous study in Seattle, Washington from 1991–1997 estimated the rate of anaphylaxis in children and adolescents to be 10.5 per 100,000 person-years [7]. The reason for this difference is likely due to differences in the definitions of anaphylaxis used. In addition, although both studies also reviewed samples of records with less specific “allergy” codes, only the Rochester study included these cases in the estimated incidence.

Although overdiagnosis of anaphylaxis can occur (due to overlap of symptoms with panic attack, hyperventilation, vasovagal episode, etc.), underdiagnosis is more common as an episode may not be recognized due to the absence of cutaneous findings or misinterpretation of nonspecific signs (eg, confusion, nausea, dyspnea). Underreporting and miscoding can also lead to an underestimation of prevalence [5, 8]. Of the approximately 12.4 million allergy-related emergency department visits from 1993–2004, only 1 % received the diagnosis of anaphylaxis [9]. Several studies have shown that anaphylaxis is often miscoded or misclassified, with 21 %–57 % of food allergy or anaphylaxis cases coded with less specific allergy or anaphylaxis codes (eg, unspecified allergy) [6–8]. As the percentage of cases identified from review of nonspecific diagnoses was similar in the Rochester study (25 %), and the Seattle study (21 %), the frequency of miscoding appears to be similar in both adults and children.

## Anaphylaxis Triggers

Across all age groups, the most common triggers for anaphylaxis are ingested foods (33 %), insect stings (19 %), and medications (14 %) [6]. Less common triggers include cats, latex, cleaning agents, environmental allergens, and exercise. For about a quarter of cases, the trigger is unknown. In children, food-induced anaphylaxis is the most common trigger and accounts for 37 %–85 % of cases, whereas insect bites/stings account for 5 %–13 % and medications account for 5 %–12 % [10–12]. Despite differences between studies, food allergy is clearly the most common cause of anaphylaxis in children.

In children, the most common food allergens are milk products (19 %–29 %), peanuts (9 %–36 %), tree nuts (9 %–19 %), eggs (5 %–22 %), shellfish (4 %–17 %), and fruits and vegetables (9 %) [10, 13, 14••, 15•]; regional differences most likely account for the differences between studies. In the United States, shellfish is the most common food allergen in persons aged ≥5 years, whereas eggs, fruits, peanuts, and tree nuts are more common in those aged <5 years [8]. The most common medication allergens are antibiotics (67 %) [10, 12].

Data suggest that the prevalence of food allergy is increasing. An analysis of multiple United States national surveys showed that food allergy in school-aged children increased from 3.3 % in 1997 to 3.9 % in 2007 [16]. In a recent United States survey of 38,480 children, the prevalence of food allergy was 8 %, with 38.7 % of those having a history of severe reactions [14••].

Information regarding the prevalence of fatal allergic reactions is limited. In an Australian study, the causes of anaphylaxis fatalities were drugs or probably drugs (58 %), insect bites/stings (18 %), undetermined (13 %), food (6 %), and other (5 %) [17]. Although most admissions for food-induced anaphylaxis occurred in children less than 5 years of age, all food-induced anaphylaxis fatalities were in patients between 8 and 35 years of age. Similarly, although insect sting-induced admissions peaked between 5 and 9 years of age, most insect sting-induced anaphylaxis deaths occurred between 35 and 84 years of age. Based on extrapolation of data from a United States-based population study, it is estimated that there are about 150 deaths annually due to food-induced allergic reactions [18]. In this study, fatalities occurred in patients aged 2 to 33 years, with 9 % in children less than 7 years of age and 53 % in teenagers.

In addition to these IgE-mediated triggers of anaphylaxis, other causes of anaphylaxis that should be considered include: galactose alpha-1,3-galactose, a carbohydrate contained in red meat that was recently described to cause anaphylaxis [19]; non-IgE-triggered mechanisms, such as through the complement and coagulation pathways initiated by oversulfated chondroitin components in heparin [20]; reactions in patients with mastocytosis and mast cell disorders; and idiopathic anaphylaxis [21••].

## Diagnosis and Management

Recommendations for the management of anaphylaxis are predominantly based on expert opinion and consensus. The AAAAI/ACAAI/JCAAI practice parameter and the World Allergy Organization guidelines provide an evidence-based approach to diagnosis and management of anaphylaxis [3••, 21••]. In addition, guidelines published by the National Institute of Allergy and Infectious Diseases (NIAID) for the diagnosis and management of food allergy [2••] provide a paradigm for the acute management of food-induced anaphylaxis, which is similar to treatment of anaphylaxis as a result of other causes. A version of this guideline that focuses on the pediatric population is also available [22].

Guidelines for both adults and children stress rapid diagnosis as being key to optimal management [2••, 3••, 21••, 22]. Anaphylaxis affects multiple organs, including the skin, respiratory tract, gastrointestinal tract, cardiovascular system, and central nervous system [23]. Signs and symptoms of anaphylaxis for adults and children are summarized in Table 1 [3••, 11]. Although cutaneous symptoms predominate in adults, the primary presenting symptoms in children are respiratory in nature (e.g., wheezing, shortness of breath) [11]. In addition, cardiovascular symptoms tend to be less common in children (17 %) than in adults (30 %–35 %) [3••, 11]. This could be due to increasing age and comorbid disease in adults; however, it could also be due to differences in the prevalence of triggers between adults and children. Food related causes, which tend to cause respiratory tract involvement, are more common in children whereas medication and venom causes, which tend to cause cardiovascular reactions, are more common in adults [10].

**Table 1:** Signs and symptoms of anaphylaxis

Symptoms	All ages [3••]		Children [11]	
	Clinical features	Frequency	Clinical features	Frequency
Respiratory	Dyspnea, wheeze	45 %–50 %	Difficulty/noisy breathing	83 %
	Upper airway angioedema	50 %–60 %	Wheeze	59 %
	Rhinitis	15 %–20 %	Cough	33 %
			Swelling tongue	13 %
			Swelling/tightness in throat	11 %
			Difficulty talking/hoarse voice	13 %
Cutaneous	Urticaria, angioedema	85 %–90 %	Urticaria	72 %
	Flushing	45 %–55 %	Angioedema	55 %
	Pruritus without rash	2 %–5 %	Pruritus	11 %
Gastrointestinal	Nausea, vomiting, diarrhea, cramping pain	25 %–30 %	Vomiting, diarrhea, abdominal cramps	29 %
Cardiovascular	Dizziness, syncope, hypotension	30 %–35 %	Hypotension, pale and floppy, impaired/loss of consciousness, collapse	17 %

Typically, exposure to the triggering allergen is followed by the rapid development of symptoms over minutes to several hours. In both adults and children, the time course of the reaction may be uniphasic (occurring immediately after exposure and resolving with or without treatment in minutes to hours), biphasic (recurring after the apparent resolution of initial symptoms, usually about 8 h after the first reaction), or protracted (persisting for hours or days following the initial reaction) [2••, 22]. Early recognition of signs and symptoms, timing of the reaction, and existence of comorbid conditions and concomitant factors can aid in diagnosis [2••].

The development of diagnostic criteria represents an important advancement in anaphylaxis management, and it is estimated that these criteria enable health care providers to identify about 95 % of cases [1]. Nevertheless, accurate diagnosis in children presents challenges. This is partially due to the inability of children to accurately describe their symptoms [24], and the lack of cutaneous symptoms in about 18 % of cases [10].

Parents and caregivers of children with food allergies are often unable to recognize and manage anaphylaxis. In studies evaluating parents, only 48 % of parents could identify more than one symptom that would require use of epinephrine [25], and only 43.5 % reported receiving education regarding their child’s food allergy and management of his/her reactions [26]. Venues where children are supervised or receive care, such as schools and child care centers, also need to be prepared to recognize and manage anaphylaxis. In a study of anaphylactic events in 48 Massachusetts public school districts, of 114 subjects who received epinephrine in the school setting (46 % were of elementary age), school personnel were unaware that the individual had a life-threatening allergy in 24 % of cases [27].

Physicians may also be unable to correctly diagnose food-induced anaphylaxis because of inadequate knowledge of food allergies. In a case-based survey of 419 pediatricians without specialized allergy training, only 56 % of respondents could appropriately recognize and treat food-induced anaphylaxis [28]. An analysis of referrals to a pediatric allergy clinic found that only 34.5 % of food allergy cases were accurately diagnosed [26]. These observations underscore a need to educate physicians and families on recognition of anaphylaxis and improve competence in recognizing this potentially fatal condition.

## Treatment

### First-Line Treatment

Evidence-based guidelines recommend the prompt administration of epinephrine as first-line treatment for an anaphylactic episode [2••, 3••, 21••]. Timely administration of epinephrine can be life-saving and help delay the progression of a life-threatening reaction so that medical attention can be provided [29]. Table 2 outlines the basic steps for management of anaphylaxis [21••].

**Table 2:** Basic management of anaphylaxis

1. Have a written emergency protocol for the recognition and treatment of anaphylaxis and rehearse it regularly.
2. Remove exposure to the trigger if possible (eg, discontinue an intravenous diagnostic or therapeutic agent that seems to be triggering symptoms).
3. Assess the patient’s circulation, airway, breathing, mental status, skin, and body weight (mass).
Promptly and simultaneously, perform steps 4–6
4. Call for help: resuscitation team (hospital) or emergency medical services (community) if available.
5. Inject epinephrine (adrenaline) intramuscularly in the mid-anterolateral aspect of the thigh (0.01 mg/kg of a 1:1000 (1 mg/mL) solution), maximum of 0.3 mg for children (0.5 mg for adults); record the time of the dose and repeat it in 5–15 min, if needed. Most patients respond to 1 or 2 doses.
6. Place the patient in a position of comfort and elevate the lower extremities. (Note: in adults, fatality can occur within seconds if the patient stands or sits suddenly. It is not known if this also applies to children.)
7. When indicated, give high-flow supplemental oxygen (6–8 L/min) by face mask or oropharyngeal airway.
8. Establish intravenous access using needles or catheters with wide-bore cannulae (14–16 gauge). When indicated, give 1–2 L of 0.9 % (isotonic) saline rapidly (e.g., 10 mL/kg in the first 5–10 min to a child).
9. When indicated at any time, perform cardiopulmonary resuscitation with continuous chest compressions.^a^
In addition,
10. At frequent, regular intervals monitor patient’s blood pressure, cardiac rate and function, respiratory status, and oxygenation (monitor continuously, if possible).

^a^Resuscitation guidelines recommend initiating cardiopulmonary resuscitation with chest compressions only (hands-only), before giving rescue breaths. In children, the rate should be at least 100 compressions/min at a depth of 5 cm (4 cm in infants)

(Adapted from Simons et al. [21••])

In children, the recommended dose of epinephrine is 0.01 mg/kg of a 1:1000 (1 mg/mL) solution via intramuscular injection into the mid-anterolateral thigh [3••]. Autoinjector dosing for epinephrine is 0.15 mg for children who weigh 10–25 kg and 0.3 mg for those who weigh >25 kg [22]. Repeated dosing of epinephrine is recommended for suboptimal response or symptom progression [2••, 3••]. Intravenous infusion of epinephrine or intravenous bolus should be considered if shock has developed or cardiac arrest is imminent [22].

According to a consensus statement from the AAAAI regarding the management of anaphylaxis in patients with a previous anaphylactic reaction in the child care setting, epinephrine should be given at the start of any reaction occurring subsequent to contact with a known or suspected allergen [30]. Calling for medical help and concurrent elimination of additional allergen exposure is also recommended [2••]. Although it is generally recommended to place the patient in a recumbent position with lower extremities elevated, individuals who are experiencing respiratory distress, which is common in children, and/or vomiting should instead be placed in a comfortable position with lower extremities elevated [21••]. If possible, supplemental oxygen and fluid resuscitation should be provided [31].

Epinephrine has alpha- and beta-adrenergic properties through which it increases blood pressure, prevents and relieves hypotension and shock, decreases upper airway obstruction (e.g., in the larynx), decreases urticaria and angioedema, and decreases wheezing [21••]. Patients may experience self-limiting effects after epinephrine administration, such as pallor, tremor, anxiety, palpitations, dizziness, and headache [21••]. In both adults and children, significant adverse effects, such as ventricular arrhythmias, hypertensive crisis and pulmonary edema, can occur after an overdose of epinephrine by any route of administration, although typically they are reported after intravenous dosing (e.g., rapid infusion, bolus administration, dosing error due to administration of concentrated solution appropriate for intramuscular injection). Misunderstanding about the correct dose and route of epinephrine administration in hospital and emergency department settings can lead to serious cardiovascular complications from overdose [32•]. In infants especially, it is important to use caution when calculating and drawing up an epinephrine dose, to stay vigilant for changes in vital signs, and to ensure use of age-appropriate blood pressure norms [21••].

### Epinephrine Auto-Injectors

Epinephrine auto-injectors (EAIs) are the cornerstone of treatment in the first-aid management of anaphylaxis in the community setting. For allergic reactions occurring in the community setting, it is recommended to administer the patient’s EAI without delay [31]. A second dose can be administered after 5–10 min based on patient status.

EAIs are often prescribed because of their ease of use and ability to rapidly produce peak epinephrine concentrations following intramuscular injection [33, 34]. Children at risk for anaphylaxis may need to carry 2 doses of epinephrine for several reasons: the first dose may not be administered effectively; symptoms may persist despite a successful first injection; or the patient may experience biphasic anaphylaxis. In a study of children with multiple food allergies, 19 % of food-induced anaphylactic reactions required ≥2 doses of epinephrine and 6 % of reactions required ≥3 doses [35]. Existing EAIs, EpiPen (Dey Pharma, L.P., Basking Ridge, NJ), TwinJect (Shionogi Inc, Florham Park, NJ), Adrenaclick (Shionogi Inc.), Anapen (Lincoln Medical Ltd, Salisbury, Wiltshire, UK), and Jext (ALK Abelló Ltd, Reading, Berkshire, UK) are available in 2 pre-set doses of epinephrine (0.15 and 0.3 mg). It is important to note that at the time this review was written, Twinject was no longer manufactured in the United States and Adrenaclick was not marketed anymore.

While EAIs have significantly improved emergency care of anaphylactic reactions, there are several limitations with the devices currently available. One of the primary limitations is their symmetrical, pen-like appearance, which can result in accidental needle sticks [35]. From 1994–2007, prior to the redesign of the EpiPen, a total of 15,190 cases of unintentional injections from EAIs were reported with the number of reports increasing significantly (*P* < 0.001) annually across all age groups [24]. In addition to adverse effects such as local ischemia of the digit [36•], accidental finger injections may also result in partial or complete loss of the epinephrine dose for the person having an anaphylactic episode, known as the “lost dose hazard”. The symptoms and signs of local ischemia reported include pain or numbness, pallor, cyanosis, hypothermia, absence of sensation or hyperesthesia, and weak or absent pulse, as well as skin peeling, sensory loss, neuropraxia, and protracted ischemia reperfusion pain. It is to be noted that none developed tissue necrosis and that overall epinephrine is associated with a good safety profile and most adverse events related to unintentional injection of epinephrine resolve without additional complications within 2–24 h with or without treatment [36•]. Studies indicate that only 25 %–55 % of patients carry their EAI with them at all times, as recommended [29, 37•]. This may be due, at least in part, to the bulky size and shape of currently available EAIs. It is important to note that the second-dose feature available in some types of EAIs (i.e., TwinJect; Shionogi Inc) requires handling an exposed, used needle [38••]. In the school setting, such injectors should be disposed of after the first dose has been used to reduce the risk of needle-stick injury. Therefore, a separate unit should be used if a second dose is required [38••]. The pre-set, fixed dose ranges (0.15 and 0.3 mg) of currently available auto-injectors can be a limitation in the pediatric setting as the 0.15-mg dose may be too strong for infants and toddlers weighing <15 kg, and the 0.3-mg dose may be subtherapeutic for children weighing >30 kg, particularly those who are overweight or obese [39]. However, data to indicate what dose is correct, inadequate, or adequate in children are currently lacking and further studies are needed.

Current EAIs have a needle length of 1.27 cm for the 0.15 mg dose EAI and 1.58 cm for the 0.3 mg dose EAI, which may be too short to penetrate the subcutaneous tissue to achieve intramuscular injection in children who are overweight or obese [40•]. Therefore, with currently available EAIs, it is important to recognize that children who are overweight or obese may be inadvertently receiving a subcutaneous injection, which will result in delayed epinephrine absorption and a lower plasma concentration of epinephrine [33]. To address this problem, additional research on needle length is necessary. The United States Food and Drug Administration has issued guidance to the medical device industry regarding the incorporation of human-factor engineering principles into improving the design and safety of medical devices [41]. Much of this guidance involves identifying and preventing user-related hazards. As this guidance becomes operational, it is hoped that further research will be undertaken to address the discussed unmet needs.

In order for EAIs to be effective, they must be used correctly and in a timely manner. However, there is a lack of patient/caregiver education and ongoing skill-retention regarding symptom recognition and proper epinephrine administration in several settings [25, 42]. In a survey that assessed patient/parent knowledge and usage of EAIs, 86 % of families indicated they kept the epinephrine device with them at all times, yet only 71 % of participants had their device with them at their office visit, and only 32 % of participants could correctly demonstrate how to use the EAI [29]. Furthermore, 10 % of participants possessed devices that were expired, leaving just 55 % of families with unexpired epinephrine on hand at the time of the survey [29]. School-aged children (aged ≥5 years) were less likely than younger children to have their EAI with them when eating lunch (25 % vs 42 %) or a snack (28 % vs 37 %) [37•]. In a recent study of 14,677 patients who filled an initial prescription for an EAI only 46 % ever refilled the prescription (63 % for children), and only 11 % refilled it at all the expected refill times [43]. It has been shown that parental empowerment and training on the use of an EAI significantly (*P* ≤ 0.05) correlates with greater parental comfort with administration [44], and EAI-training improves the odds of having an EAI readily available [37•]. In a quality-improvement project at the Children’s Mercy Hospital (Kansas City, MO) in which data from 277 patients at-risk for anaphylaxis was collected, less than half (44 %) of the patients had their EAI devices with them. The most common reason cited (47%−56%) for not having the EAI device during their visit was not realizing they had to carry it at all times (other reasons included forgot, expired, lost, etc.). At the initial visit, only about 3 out of 5 caregivers were able to correctly perform all the steps to use the device. Our study reveals that a systematic and periodic process of screening, education, and re-education on carriage, and knowledge of use of this device is needed. This process is now routine and mandatory in our clinics for all patients at risk for anaphylaxis.

Preparedness for appropriately treating anaphylactic reactions is suboptimal in child care centers. In a survey of 42 child care centers tending children aged 6 months to 6 years in the suburbs of Chicago, only 24 % of center directors initially stated that they would administer EpiPen for a severe allergic reaction and only 55 % had trained staff for this emergency [13]. An assessment of the long-term effectiveness of an allergy seminar identified a need for recurring anaphylaxis education among child care providers [42]. Although 77 % of child care center directors could demonstrate EAI technique at 4 weeks post-training, knowledge decreased to 48 % at 6 months, and 31 % at 1 year.

Gaps in anaphylaxis management extend to clinicians as well, including incomplete understanding of how and when to use EAIs, and inadequate provision of, or arrangement for patient education. An assessment of 29 attending pediatricians found that only 24 % provided families with written indications for use of the EAI, only 21 % could correctly demonstrate technique, and 14 % of pediatricians familiar with EpiPen/EpiPen Jr incorrectly thought the device should be refrigerated [29]. In a separate survey of food-allergic individuals or caregivers (*n* = 1887), only 58.7 % of respondents reported receiving training from the prescriber on the use of auto-injectors [45]. Further, 86.6 % of participants did not recall receiving oral counseling during dispensation of EAI at the pharmacy [45]. These findings highlight the need for all health care professionals to become comfortable with EAI usage and to educate patients and their families appropriately. Health care professionals should also remind patients that EAIs expire 1 year after dispensing [2••], and need to be stored at room temperature [38••].

Physicians can help strengthen parental education by appropriate referral to an allergist for children with anaphylaxis [3••]. Just 1 visit to an allergy clinic has been reported to improve parental knowledge of allergen avoidance, management of allergic reactions, and use of an EAI [26]. Physicians should also develop emergency action plans (e.g., http://www.foodallergy.org/files/FAAP.pdf) for children with food allergies [3••]. An e-mail survey of 1885 individuals who had survived anaphylaxis or had been responsible for someone who had survived anaphylaxis, reported that 62 %–64 % of participants did not have a plan of action readily available [46]. Food-allergy emergency action plans are essential in the school setting also. A recent survey of elementary and middle school nurses from 43 schools in South Carolina found food-allergy emergency action plans in place in only 44 % of schools [47]. As an added consideration, Bansal et al. reported that most child care centers (98 %; *n* = 41) do not have medications on hand to treat an allergic reaction unless provided by the caregiver [13]. Thus, in addition to establishing a standard anaphylaxis-management protocol, state/federal policy must permit schools to have a supply of epinephrine on hand for general use [27, 38••].

In summary, in the community, prompt administration of epinephrine via an EAI is crucial. Ongoing education is required to ensure patients and child care providers always have an unexpired EAI with them, know how to correctly use their EAI, and know what to do in case of a severe allergic reaction. In addition, health care professionals need to become comfortable with EAI usage and provide appropriate education for patients/caregivers.

### Second-Line Treatment

Second-line treatment options in the outpatient setting include β_2_-agonists, antihistamines, and glucocorticoids. These agents may be administered in a hospital-based setting, along with vasopressors, glucagon, and activated charcoal [2••, 3••, 48]. Although commonly used, data supporting the role or effectiveness of second-line treatment options in the management of anaphylaxis are limited [49, 50]. Hence, these second-line treatments should be considered only as adjunct therapy to epinephrine.

## Preventive Measures

Preventive measures may reduce the risk of anaphylaxis in susceptible individuals. Anaphylaxis education should begin prior to discharge from the health care facility and include a prescription for an EAI, education on technique, recommendation of a medical identification bracelet or wallet card, and referral to an allergy/immunology specialist for assessment of triggers. Anaphylaxis triggers should be identified by taking a detailed history, and be confirmed when possible by using allergen skin tests or serum allergen specific IgE levels [21••].

Avoidance of suspected triggers or co-triggers is key to the management of anaphylaxis; however, this approach is often unsuccessful due to inadequate patient education and understanding of allergen avoidance, and a lack of awareness of triggers, particularly during a first episode [51, 52]. Physicians should provide families of allergic children with detailed information regarding relevant triggers and how to avoid them. In addition, the Food Allergy and Anaphylaxis Network website contains information on food allergen avoidance, among other issues, and may be a useful resource for families and physicians (http://www.foodallergy.org).

In addition to avoidance, the guidelines recommend patients should receive a prescription for an EAI and referral to an allergy/immunology specialist [3••]. However, retrospective studies report low compliance with these guidelines in both adults and children, although concordance with recommended care was somewhat better among patients who were admitted to the hospital (59 % prescribed EAI; 35 % referred to allergist) [53]. In children considered to have food-induced anaphylaxis, 51 %–63 % received an EAI prescription and 24 %–33 % were referred for follow-up [14••, 15•]. Among those admitted to hospital, 94 % were prescribed an EAI and 69 % were referred to an allergist [15•]. These data indicate that many patients are not receiving basic tools to prevent or manage subsequent anaphylaxis events.

Several other preventive measures are available for allergen triggers, such as immunotherapy for insect bites/sting reactions, avoidance of the drug or therapeutic substitution with a non–cross-reacting medication, and desensitization protocols for drug allergy. Patients with frequent episodes of idiopathic anaphylaxis may benefit from prophylactic treatment with a systemic glucocorticoid and an H1-antihistamine [3••] or prophylactic omalizumab injections [21••].

Experimental approaches to prevent food-allergy reactions include immunotherapy [54, 55], anti-IgE therapy [56], and possibly early introduction of solids and allergenic food contrary to past infant-feeding guidelines [57]. Preclinical data hint at the potential efficacy of Chinese herbal medicine [58] and probiotic supplementation [59] in food allergy prevention. However, studies are needed to investigate the effectiveness and safety of the above approaches for preventing anaphylaxis in children with food allergy.

## Conclusions

Numerous gaps in the diagnosis and management of anaphylaxis in children exist at both the physician and caregiver/patient level. The key to successful management involves recognition of populations at risk (e.g., children with food allergies) and rapid diagnosis with early initiation of effective evidence-based therapy. Ongoing education is required to improve the ability of physicians and families to recognize anaphylaxis in children. Epinephrine is the recommended first-line treatment for anaphylaxis in both inpatient and outpatient settings, and should be administered promptly upon recognition of signs and symptoms. Although second-line treatments are available, they should be considered only as adjunct therapy to epinephrine. Ongoing education is required to ensure patients and child care providers understand the importance of always having an unexpired EAI with them, knowing how to correctly use their EAI, and knowing what to do in case of a severe allergic reaction.

Epinephrine auto-injectors allow for the prompt administration of epinephrine in the community setting and must be prescribed to all patients at risk. Advances in the design of these devices to improve safety and convenience of carriage and use, may further aid in successful management of children with anaphylaxis.
